# 9-*O*-butyl-13-(4-isopropylbenzyl)berberine, KR-72, Is a Potent Antifungal Agent That Inhibits the Growth of *Cryptococcus neoformans* by Regulating Gene Expression

**DOI:** 10.1371/journal.pone.0109863

**Published:** 2014-10-10

**Authors:** Soohyun Bang, Hyojeong Kwon, Hyun Sook Hwang, Ki Duk Park, Sung Uk Kim, Yong-Sun Bahn

**Affiliations:** 1 Department of Biotechnology, Yonsei University, Seoul, Republic of Korea; 2 Center for Neuro-Medicine, Brain Science Institute, Korea Institute of Science and Technology, Seoul, Republic of Korea; 3 Industrial Bio-materials Research Center, Korea Research Institute of Bioscience and Biotechnology, Daejeon, Republic of Korea; Research Institute for Children and the Louisiana State University Health Sciences Center, United States of America

## Abstract

In this study we explored the mode of action of KR-72, a 9-*O*-butyl-13-(4-isopropylbenzyl)berberine derivative previously shown to exhibit potent antifungal activity against a variety of human fungal pathogens. The DNA microarray data revealed that KR-72 treatment significantly changed the transcription profiles of *C. neoformans*, affecting the expression of more than 2,000 genes. Genes involved in translation and transcription were mostly upregulated, whereas those involved in the cytoskeleton, intracellular trafficking, and lipid metabolism were downregulated. KR-72 also exhibited a strong synergistic effect with the antifungal agent FK506. KR-72 treatment regulated the expression of several essential genes, including *ECM16*, *NOP14*, *HSP10* and *MGE1*, which are required for *C. neoformans* growth. The KR-72-mediated induction of *MGE1* also likely reduced the viability of *C. neoformans* by impairing cell cycle or the DNA repair system. In conclusion, KR-72 showed antifungal activity by modulating diverse biological processes through a mode of action distinct from those of clinically available antifungal drugs such as polyene and azole drugs.

## Introduction

Over the past decades, fungal pathogens have emerged as a global threat to the ecosystem, including humans [1], [2]. In particular, systemic mycoses caused by primary or opportunistic fungal pathogens pose significant medical problems to public health, mainly due to the growing number of aging persons, and immunocompromised individuals who undergo solid organ transplantation and anticancer-chemotherapy, or have HIV-infection. Nevertheless, only a limited number of antifungal drugs are clinically effective because fungi and mammals share most cellular features, with a few exceptions. One exception is ergosterol, a sterol that plays a role in fungal membrane integrity and plasticity. The common antifungal drugs include a polyene class of drugs (e.g., nystatin and amphotericin B) that bind to ergosterol and form pores through the membrane, and the azole (e.g. fluconazole) and allylamine (e.g., terbinafine) class of drugs that respectively inhibit 14-α-demethylase (Erg11) and squalene epoxidase (Erg1) required for ergosterol synthesis [3]. Since both polyene and azole drugs respectively cause nephrotoxicity and hepatotoxicity [3], a novel class of antifungal drugs with lower toxicity and high efficacy needs to be identified and clinically developed.

Previously, we have synthesized novel 13-(4-isopropylbenzyl)berberine derivatives, which exhibit a broad-spectrum of antifungal activities [4], [5]. Berberine is an isoquinoline alkaloid isolated from Korean and Chinese medicinal plants that inhibits the growth of a wide range of *Candida* species [6]. Among the berberine derivatives, 9-*O*-butyl-13-(4-isopropylbenzyl)berberine, also known as KR-72, showed the most potent antifungal activity against *Cryptococcus* and *Candida* species (minimum inhibition concentration (MIC) = 0.25–8 mg/L). Therefore, it has been considered as a potential antifungal drug candidate for the treatment of various fungal diseases.

Despite KR-72 showing potent antifungal activity, its mode of action and the physiological impacts of the drug on fungal metabolism remain to be fully elucidated. Herein, KR-72-responsive genes were identified through DNA microarray-based transcriptome analysis, and their functions were characterized using reverse genetics approaches in *C. neoformans*, which causes fatal meningoencephalitis in humans and is responsible for more than 600,000 deaths annually worldwide [7].

## Results and Discussion

### DNA microarray-based transcriptome analysis for the identification of KR-72 responsive genes in *C. neoformans*

To elucidate the mode of antifungal action for KR-72, we monitored the transcriptome profile of fungal cells treated with KR-72 via DNA microarray analysis. For this purpose, we used DNA microarray platforms available in the *C. neoformans* var. *grubii* H99 strain as a fungal pathogenic model organism. We treated the H99 strain with 1 mg/L KR-72 and isolated total RNA after a 30 min or 60 min incubation period. For each time point, 3 independent RNA samples were prepared as biological replicates to obtain significant statistical results.

**Figure 1:**
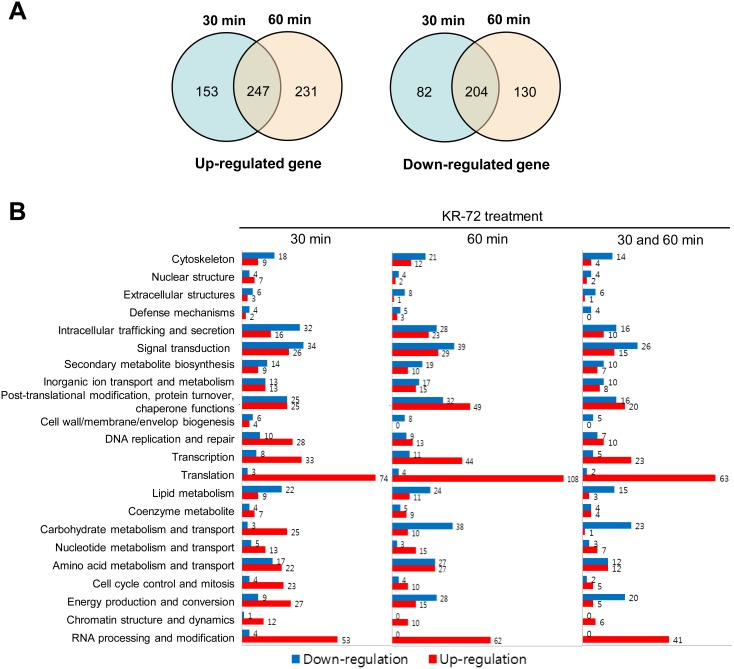
Functional categories of KR-72-responsive genes in *C. neoformans.* (a) Venn diagrams showing number of upregulated (left diagram) and downregulated *C. neoformans* genes (right diagram) with 30 min and 60 min treatment of KR-72. The number was counted only for genes whose expression levels were significantly changed (ANOVA, *P*<0.05). (b) Functional categories of KR-72 responsive genes in *C. neoformans*. Among the KR-72 responsive genes, genes whose expression was changed more than 1.5-fold were functionally categorized based on the COG (eukaryotic Cluster of Orthologous Groups of proteins, http://www.ncbi.nlm/nih.gov/COG/) functional description. The red and blue bars indicate the number of up-regulated and down-regulated genes by KR-72, respectively.

The DNA microarray analysis revealed that transcriptome profiles of *C. neoformans* underwent significant changes during KR-72 treatment. After 30 min of treatment, the expression of a total of 1,671 genes was significantly altered (Table S3 in File S1). Among them, 1,014 genes exhibited more than a 1.5-fold change in expression (Tables S4–S6 in File S1), whereas 400 genes showed more than 2-fold changes (Fig. 1A). After 60 min of treatment, the expression of more genes (total 2,034 genes) was significantly affected. A total of 1,258 genes exhibited more than a 1.5-fold change in expression, while 392 genes showed more than 2-fold changes. The expression of 451 genes was regulated by both the 30 min and 60 min KR-72 treatments (Fig. 1A).

The functional categories of KR-72 responsive genes, which were classified by the KOG (eu*k*aryotic *o*rthologous *g*roup), provided insight on the mode of action of KR-72 (Fig. 1B). Notably, the genes involved in amino acid transport, protein translation, and post-translation modifications were the most overrepresented, which suggested that KR-72 affects protein synthesis and modification. In particular, a number of genes involved in translation appeared to be highly induced upon KR-72 treatment. The second important class of genes included those involved in transcription and RNA processing and modification. The expression of these genes was also highly upregulated upon KR-72 treatment. In contrast, the expression of genes involved in the cytoskeleton, intracellular trafficking and secretion, and signal transduction was significantly downregulated upon treatment with KR-72 (Fig. 1B). Interestingly, genes involved in carbohydrate metabolism and energy production/conversion were upregulated at early time points (30-min treatment) but subsequently downregulated at a later time point (60 min) (Fig. 1B). In summary, KR-72 treatment affected a plethora of essential cellular processes, which is in accordance with KR-72 exhibiting antifungal activity.

### KR-72 treatment downregulated genes involved in cell membrane/wall integrity and conferred synergistic antifungal activity with FK506 by inhibiting the calcineurin pathway

**Figure 2:**
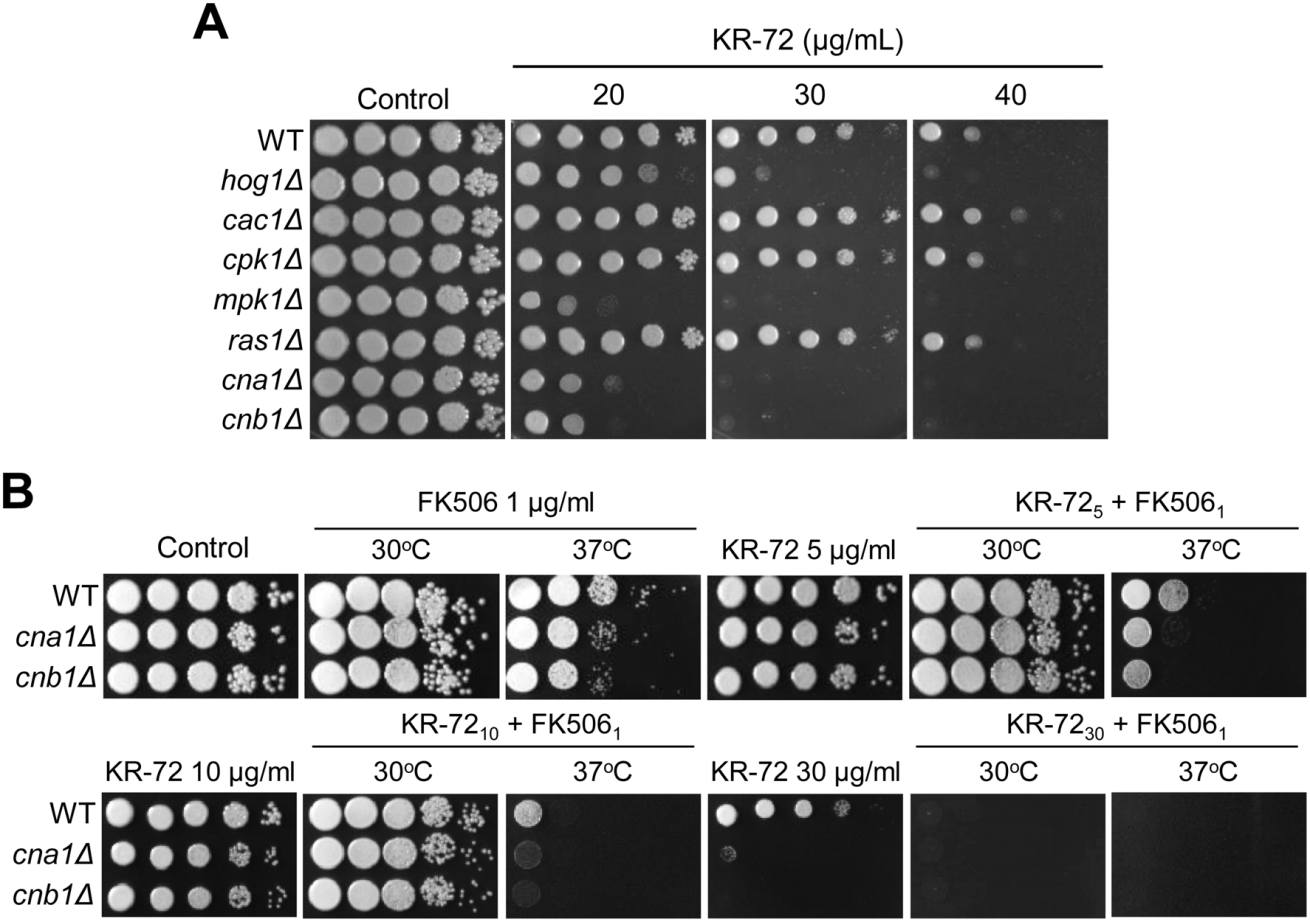
*C. neoformans* mutants defective in cell wall and membrane integrity exhibited increased susceptibility to KR-72. (a) Each *C. neoformans* strain [WT (H99), *hog1*Δ (YSB64), *cac1*Δ (YSB42), *cpk1*Δ (YSB127), *mpk1*Δ (KK3), *ras1*Δ (YSB53), *cna1*Δ (KK1), and *cnb1*Δ (KK2)] was grown overnight at 30°C in liquid YPD medium, 10-fold serially diluted (1 to 10^4^ dilutions), and spotted (3 µL) onto YPD agar containing the indicated concentrations of KR-72. Cells were incubated at 30°C for 3 days and then photographed. (b) The WT H99 strain and *cna1*Δ (KK1) and *cnb1*Δ (KK2) mutants were cultured in YPD medium at 30°C for 16–20 h, 10-fold serially diluted (1 to 10^4^ dilutions), and spotted (3 µL) onto YPD agar containing the indicated concentrations of KR-72 (5 [KR-72_5_], 10 [KR-72_10_], or 30 µg/mL [KR-72_30_]), FK506 (1 µg/mL, FK506_1_), or a combination of both (KR-72_5_+FK506_1_, KR-72_10_+FK506_1_, or KR-72_30_+FK506_1_). Cells were then incubated at 30°C or 37°C for 3 days and then photographed.

The microarray results demonstrated that a number of genes involved in cell wall/membrane/envelop biogenesis and cytoskeleton were downregulated upon KR-72 treatment, suggesting that the drug may affect cell membrane/wall integrity (Table 1). In addition, *PCM1*, which is predicted to encode an essential *N*-acetylglucosamine-phosphate mutase required for chitin synthesis [8], was also downregulated by KR-72 treatment (Table 1). If this hypothesis is true, KR-72 may confer higher synergistic susceptibility to *C. neoformans* mutants that have defects in cell wall or membrane integrity. For example, *C. neoformans* cells with *HOG1* (stress-activated mitogen-activated protein kinase [MAPK]), *MPK1* (a cell wall integrity MAPK), *RAS1* (small GTPase), and *CNA1/CNB1* (catalytic and regulatory subunits of the calcineurin, respectively) deletion is known to exhibit defective cell wall/membrane integrity. Among these, the *hog1Δ*, *mpk1Δ,* and *cna1Δ/cnb1Δ* mutants indeed exhibited hyper-susceptibility to KR-72 (Fig. 2A), indicating that KR-72 could destabilize the cell membrane/wall integrity in *C. neoformans*. Particularly, the fact that both *cna1Δ* and *cnb1Δ* mutants exhibited a greater sensitivity to KR-72 than the wild-type strain suggested that a combination treatment of KR-72 with FK506, which inhibits the calcineurin activity in *C. neoformans*, could be more effective in killing *C. neoformans* than a individual treatment of each drug. Supporting this hypothesis, co-treatment of *C. neoformans* with KR-72 and FK506 was much more effective in killing the fungus than each single treatment (Fig. 2B). Combination treatment of KR-72 with FK506 exhibited apparent synergistic antifungal activity at 37°C (Fig. 2B). In the checkerboard assay, the synergistic interaction between KR-72 and FK506 was evident only at 37°C (FIC index = 0.25; Table 2), but not at 30°C (FIC index = 1.5).

**Table 1:** List of *C. neoformans* genes regulated by KR-72.

KOGs Category	Up-regulated genes*	Down-regulated genes*
**RNA processing** **and modification**	*RRP7*, *SIK1*, *PRP43*, *RNA1*,*NOP10*, *MTR4*, *NOP58*,*DBP7*, *IMP4*, *NOP1*, *RCL1*,*MAK5*, *DIM1*, *PWP2*, *HCA4*,*BUD20*, *EBP2*, *UTP13*,*DBP8*, *MPP10*, *NHP2*,*ECM16*, *DBP6*, *DBP9*,*NOP4*, *RRP3*, *SPB1*, *NOP2*,*RRP5*, *DBP3*, *NOP7*, *MRT4*	
**Chromatin structure** **and dynamics**	*ELP2*, *SAS10*	
**Energy production** **and conversion**	*DIC1*, *CYC3*, *RIM2*,*FDH1*, *AIF1*	*MAE1*, *ATG26*, *ADH4*, *PPX1*, *YHB1*, *GUT2*, *CYB2*, *SDH2*, *FDH1*
**Cell cycle control** **and mitosis**	*LUG1*, *SDA1*	*NPR1*, *CUL3*
**Amino acid** **metabolism** **and transport**	*AVT1*, *PRS3*,*MET13*, *SPT16*, *HIS7*,*FSH1*, *NEW1*	*PDC1*
**Nucleotide metabolism** **and transport**	*ADE5/7*, *PRS3*,*RNR3*, *ADE4*, *URA7*	*GUD1*
**Carbohydrate** **metabolism** **and transport**		*YEF1*, *ATG26*, *FBP1*, *TPS1*,*NTH1*, *TPS1*, *GUT1*, *TPS2*,*SUC2*, *AQY1*, *PCM1*, *RIM11*,*HXK2*
**Coenzyme metabolism**	*MET7*, *MTD1*	*PDC1*, *HEM13*
**Lipid metabolism**		*NCR1*, *OLE1*, *YPC1*, *DGA1*,*OSH1*, *CAT2*, *FAA1*
**Translation**	*MRPL51*, *MRPL10*, *MRP2*,*RRP6*, *RML2*, *EMG1*,*GCD14*, *TIF6*, *SSF2*, *IFM1*,*SIK1*, *PUS4*, *PUS1*, *NOP14*,*NOP58*, *MEF1*, *TRM2*,*SMM1*, *RLP24*, *MRI1*,*NEW1*, *BRX1*, *ECM16*,*NOC4*, *ERB1*, *NOC3*,*NMD3*, *PUF6*, *NIP7*, *DPH5*, *MAK21*	*HBS1*, *SSD1*
**Transcription**	*RPB5*, *ELP2*, *RPO26*, *BFR2*,*RPO41*, *RPO31*, *RPA190*,*RPA49*, *RPB10*, *NOP13*,*RPA135*, *RPA12*, *NSR1*, *MAK21*	
**Replication and repair**	*ATP23*, *MCM3*, *POL30*,*PAP2*, *MCM2*, *RPO41*	*YSA1*, *RAD1*, *DNL4*, *MAG1*,*NPY1*
**Cell** **wall/membrane/envelop** **biogenesis**		*AIM25*, *GPI12*
**Post-translational** **modification,** **protein turn** **over,** **chaperon function**	*KAE1*, *CYC3*, *MAP1*, *AFG2*,*RIX7*, *PNO1*, *MGE1*, *HSP10*,*URM1*	*ECM4*, *YUH1*, *VTC1*, *SEC18*,*RKR1*, *OTU1*, *UBA3*
**Inorganic ion transport** **t and metabolism**	*ECM17*, *ZRT1*, *PHO84*	*MMT2*, *ENA2*
**Secondary** **Metabolite** **biosynthesis**	*IDI1*, *YOR1*	*ADP1*, *ADH3*, *GUD1*
**Signal transduction**	*PTC7*, *RNA1*, *SPE2*, *PLC1*	*RCK2*, *GYP7*, *OTU1*, *LDB17*,*SCH9*, *VPS15*, *KES1*
**Intracellular** **trafficking** **and secretion**	*BFR2*, *BST1*, *TIM22*, *NOC3*	*PEP3*, *INP54*, *AIM30*, *ATG2*,*VTC1*, *VPS33*, *NPL4*, *PEP5*,*SEC3*
**Extracellular** **structures**	*ENP1*	
**Nuclear structure**	*RNA1*, *SPR4*	*NPL4*
**Cytoskeleton**	*YTM1*, *SDA1*	*CAP2*, *ABP1*, *ARP2*, *ARP3*,*ARP2*, *ARC18*, *ATG8*, *LDB17*,*SLA2*

* *C. neoformans* genes, whose orthologs exist in *S. cerevisiae* and their expression was regulated more than 2-fold by 30 min or 60 min treatment of KR-72, were listed. Putative essential genes, whose deletion is known to be lethal in *S. cerevisiae*, were underlined.

**Table 2:** *In vitro* antifungal activities of KR-72 combined with commercially available antifungal agents against *C. neoformans.*

Temp. (°C)	MIC_50_ alone (mg/L)^a^	MIC_50_ combined (mg/L)^a^	FIC index^b^
30	KR (4), FK506 (>32)	KR/FK506 (2), FK506/KR (>32)	1.5
37	KR (4), FK506 (0.01)	KR/FK506 (0.2), FK506/KR (0.002)	0.25
35	KR (4), FCZ (>2)	KR/FCZ (>4), FCZ/KR (>2)	2
35	KR (4), 5FC (>4)	KR/5FC (>2), 5FC/KR (>2)	1
35	KR (4), FDX (0.5)	KR/FDX (>4), FDX/KR (>0.5)	2

a KR, KR-72; FDX, Fludioxonil; 5Fc, 5-flucytosine; FCZ, fluconazole. MIC_50_ was determined by CLSI method. For calculation purposes, >32, >4, >2, and >0.5 were assumed to be 32, 4, 2, and 0.5. For all checkerboard assays, *C. neoformans* H99 strain was used.

b FICI≤0.5 = synergy, FICI>4.0 = antagonism, 0.5<FICI≤4.0 = no interaction, as suggested by Johnson et al. [38].

### KR-72 affected genes involved in sterol metabolism

**Figure 3:**
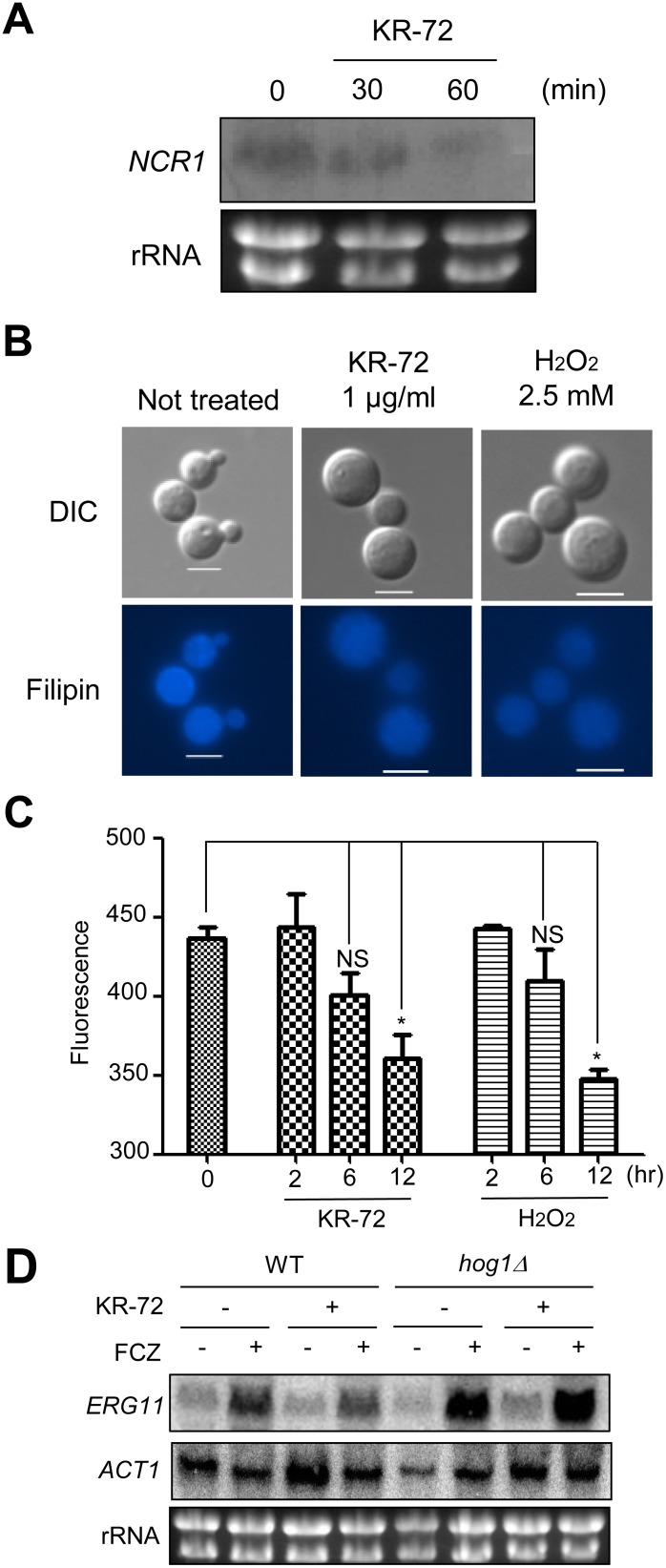
KR-72 treatment reduced *NCR1* expression and affected sterol metabolism. (a) Expression level changes of *NCR1* during KR-72 treatment (0, 30, 60 min) were measured by northern blot analysis. (b and c) To examine whether KR-72 affects the sterol transport to the cell membrane, the WT strain (H99) was exposed to 1 µg/mL KR-72 or 2.5 mM H_2_O_2_ for 60 min (b) or indicated incubation time (c), stained with 12.5 mM Filipin working solution and monitored by a fluorescence microscope (b) or fluorescence microplate reader (c) for quantitatively measuring fluorescence intensities. Bar, 10 µm. Fluorescence was calculated at OD_485–515 nm_/OD_595 nm_. Three independent triplicate experiments were performed. Standard deviations are presented as error bars. Statistical analysis was performed by Bonferroni’s multiple comparison test. Each symbol in (c) indicates the following: *, *P*<0.05; NS, not significant (*P*>0.05). (d) Expression level changes of *ERG11* by KR-72 treatment (90 min) in the WT strain (H99) and *hog1Δ* mutant were measured by northern blot analysis.

The microarray analysis also revealed that KR-72 treatment affected several genes involved in lipid metabolism. The genes downregulated by KR-72 (at both 30 min and 60 min) included *NCR1* (cholesterol transport protein), *OLE1* (fatty acid desaturase), *YPC1* (alkaline ceramidase), *DGA1* (diacylglycerol acyltransferase), *OSH1* (oxysterol-binding protein; also known as *SWH1*), *FAA1* (acyl-CoA synthetase), and *CAT2* (carnitine O-acyltransferase) (Table 1). In contrast, several *ERG* genes (*ERG13*, *ERG6*, *ERG24* and *ERG4*) were upregulated at the later time point of KR-72 treatment (60 min). Among these genes, *NCR1* and *OSH1* are involved in sterol transport. *NCR1* is the ortholog of the human Niemann Pick type C (NP-C) gene 1 (*NPC1*) and the *S. cerevisiae NP-C-*related gene 1 (*NCR1*). Mammalian cells defective in *NPC1* have defects in cholesterol transport and homeostasis [9], [10]. In *S. cerevisiae*, a dominant mutation in the sterol-sensing domain (SSD) of Ncr1 alters sphingolipid and ergosterol recycling [11]. In fact, the yeast Ncr1 is predicted to be a glycosylated transmembrane protein that is homologous to the sterol regulatory element-binding protein (SREBP) cleavage-activating protein (SCAP). We confirmed that *NCR1* expression was downregulated in response to KR-72 treatment by Northern blot analysis (Fig. 3A). *OSH1* encodes one of seven yeast oxysterol-binding proteins (Osh1–7) [12], [13] and downregulates ergosterol biosynthesis genes and performs distinct and redundant functions for cell survival [14]. Oxysterols are enzymatically or non-enzymatically oxygenated derivatives of cholesterols in mammals (ergosterols in fungi) that act as a key signalling molecules for many biological processes [15]. Osh1 localizes to both the Golgi via a pleckstrin homology (PH) domain and the nucleus-vacuole (NV) junction by its ankyrin repeat domain [16]. Interestingly, *osh1Δ* mutants exhibit *erg* mutant-like phenotypes (e.g., cold sensitivity when tryptophan levels are low), suggesting that Osh1 is involved in lipid trafficking, sterol metabolism, and homeostasis. Therefore, KR-72 treatment may perturb sterol metabolism and homeostasis, resulting in the transcriptional upregulation of some *ERG* genes as a compensatory effect.

**Figure 4:**
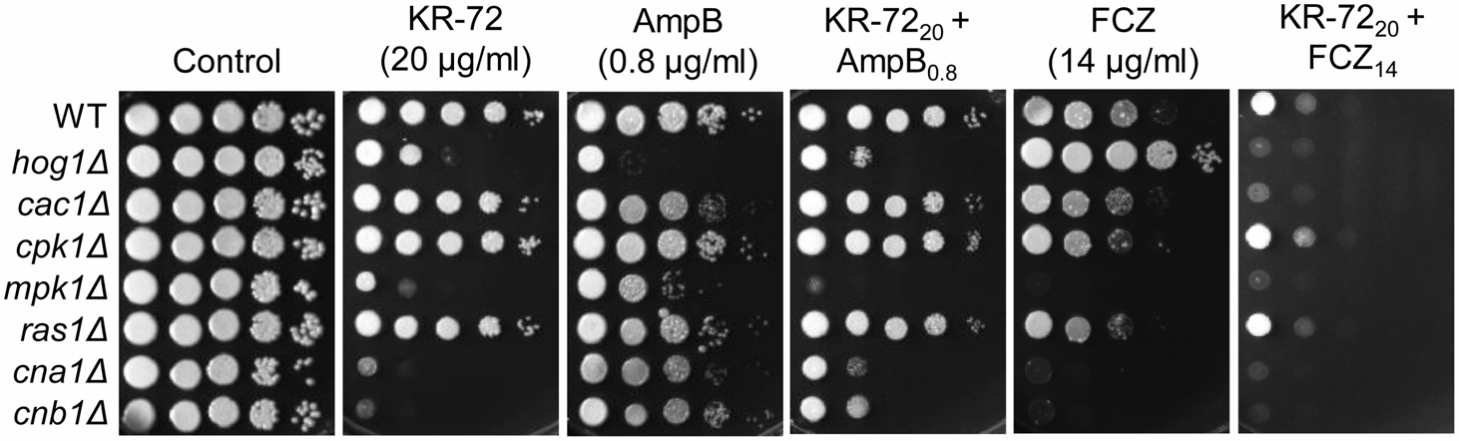
KR-72 modulated azole susceptibility in *C. neoformans.* Each *C. neoformans* strain [WT (H99), *hog1*Δ (YSB64), *cac1*Δ (YSB42), *cpk1*Δ (YSB127), *mpk1*Δ (KK3), *ras1*Δ (YSB53), *cna1*Δ (KK1), and *cnb1*Δ (KK2)] was grown overnight at 30°C in liquid YPD medium, 10-fold serially diluted (1 to 10^4^ dilutions), and spotted (3 µL) onto YPD agar containing the indicated concentrations of KR-72 (20 µg/mL, KR-72_20_), amphotericin B (0.8 µg/mL, AmpB_0.8_), fluconazole (14 µg/mL, FCZ_14_), or a combination of both (KR-72_20_+AmpB_0.8_, KR-72_20_+FCZ_14_). Cells were incubated at 30°C for 3 days and photographed.

To examine whether KR-72 affects the sterol transport to the membrane, we stained KR-72-treated cells with a sterol-binding fluorescence dye, Filipin. As a control, we also stained *C. neoformans* cells treated with H_2_O_2_, which is known to downregulate *ERG* gene expression and sterol biosynthesis [17]. Fluorescence by Filipin staining gradually decreased after KR-72 or H_2_O_2_treatment (Fig. 3B and 3C), indicating that the cell surface sterol level was decreased. Considering this phenomenon, we determined whether KR-72 treatment may affect fungal susceptibility to polyene drugs, such as amphotericin B, which directly binds to the membrane ergosterol and forms lethal pores through the cell membrane. However, amphotericin B did not show significant synergistic interaction with KR-72 (Fig. 4).

Interestingly, KR-72 treatment appeared to increase fluconazole susceptibility in the wild-type strain (Fig. 4), although the synergistic interaction between the two agents was not evident based on the FIC index (Table 2). However, KR-72 was not likely to directly target Erg11, because KR-72 treatment did not significantly increase the *ERG11* expression level while fluconazole treatment increased it (Fig. 3D). Notably, we observed that KR-72 treatment suppressed the azole resistance of the *hog1*Δ mutant (Fig. 4), which has the enhanced basal expression levels of *ERG11* [18], without significantly affecting *ERG11* induction. This indicates that azole drugs and KR-72 may have different modes of action. In summary, KR-72 downregulated genes involved in lipid metabolism and affected membrane sterol content in *C. neoformans*.

### KR-72 modulated essential genes involved in ribosomal RNA synthesis and mitochondrial chaperones to confer antifungal effects

One potential explanation for the KR-72 antifungal activity may be that this drug targets an essential protein(s), inducing the expression of the target gene as compensating effects. For example, azole drugs, which target Erg11, induce the expression of *ERG11* [19]. Therefore, we examined genes whose expression was induced more than 2-fold by KR-72 and whose orthologues were essential for the growth of *S. cerevisiae* based on the annotated *Saccharomyces* genome database (SGD). Surprisingly, 71 putative essential genes were observed to be significantly upregulated by KR-72 (underlined genes in Table 1). Among these genes, 54 genes were predicted to be involved in RNA processing/modification (27 genes), transcription (9 genes), translation (13 genes), and post-translational modification/protein turnover/chaperone functions (5 genes).

**Figure 5:**
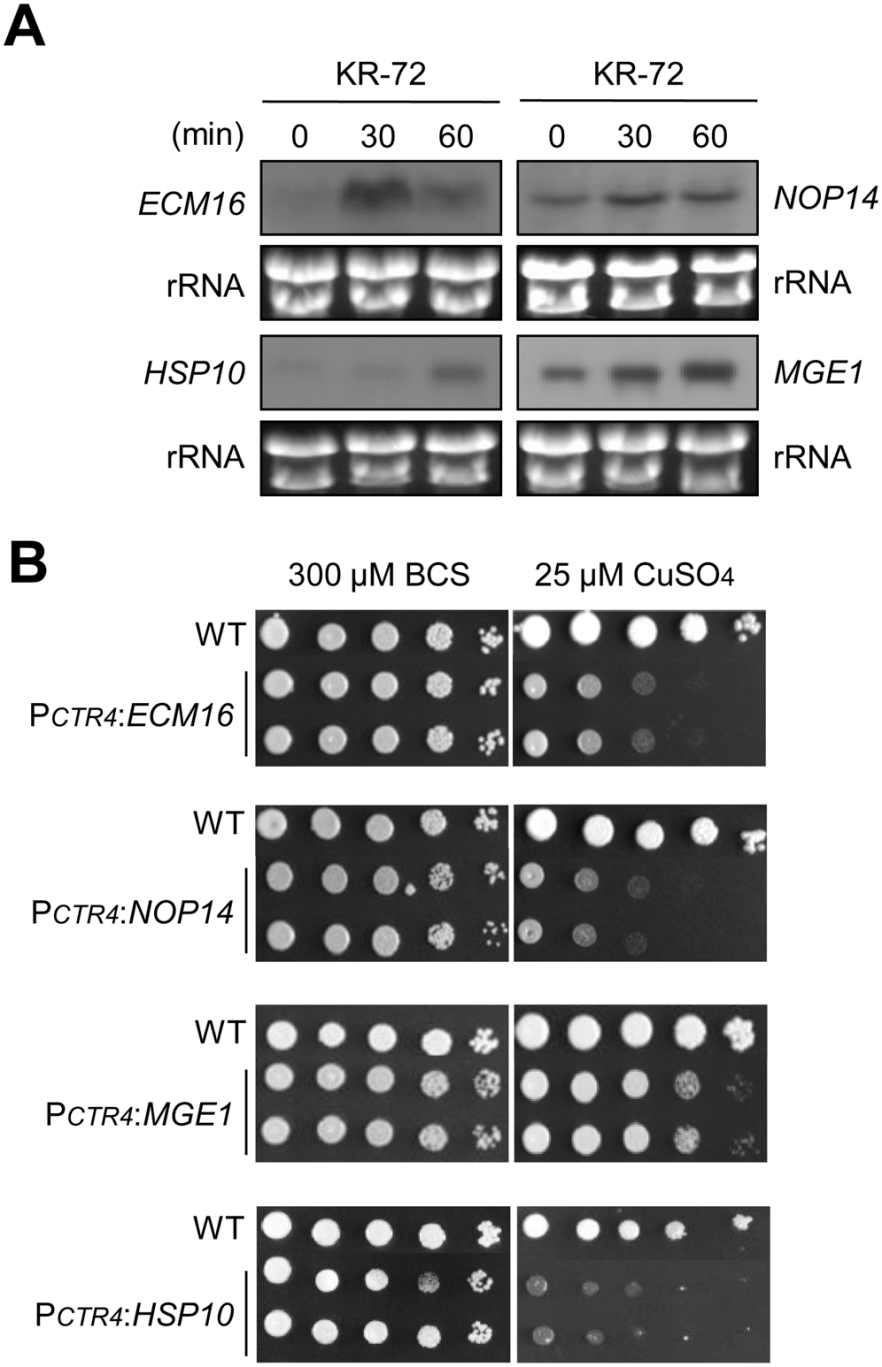
KR-72-mediated increase in the expression of essential genes, *ECM16, HSP10, NOP14,* and *MGE1*, in ribosome biogenesis and mitochondrial functions. (a) Northern blot analysis of the total RNA isolated from WT (H99) cells treated with KR-72 in YPD medium at 30°C for 0 min, 30 min, and 60 min. Each membrane was hybridized and labelled by a corresponding gene-specific probe. (b) The essentiality of *ECM16, HSP10, NOP14,* and *MGE1* in *C. neoformans*. WT H99, P*_CTR4_:ECM16* (YSB2596 and YSB2597), P*_CTR4_:HSP10* (YSB2688 and YSB2689), P*_CTR4_:NOP14* (YSB2604 and YSB2605), and P*_CTR4_:MGE1* (YSB3172 and YSB3173) strains were grown in liquid YPD medium at 30°C overnight, 10-fold serially diluted (1 to 10^4^ dilutions), and spotted (3 µL) on YNB agar media containing 200 µM BCS and 25 µM CuSO_4_. Cells were incubated at 30°C for 2 days and then photographed.

Among these upregulated essential genes, we chose four genes, *ECM16, NOP14, HSP10* (heat shock protein 10) and *MGE1*, for further functional analysis. Ecm16 is required for 18S rRNA synthesis [20], [21]. Nop14 is also essential because it is involved in ribosome biogenesis [21]. *HSP10* and *MGE1* are two essential mitochondrial co-chaperone genes. *HSP10* encodes a mitochondrial matrix co-chaperonin, which inhibits the ATPase activity of Hsp60 and is involved in protein folding and sorting in mitochondria [22]–[24]. Mge1 is a mitochondrial co-chaperonin protein that interacts with Ssc1, a mitochondrial Hsp70 [25]–[27]. We confirmed the KR-72-responsive induction of these four genes by northern blot analysis (Fig. 5A).

To further analyse the role of the four upregulated essential genes, we first examined their essentiality in *C. neoformans* growth. For this purpose, we constructed promoter replacement strains with a copper-regulated *CTR4* promoter (P*_CTR4_*:*ECM16*, P*_CTR4_*:*NOP14,* P*_CTR4_*:*HSP10* and P*_CTR4_*:*MGE1* strains), as illustrated in Fig. S1A in File S1 and described in Materials and Methods. For verification of their essentiality, more than two independent promoter replacement strains were constructed and confirmed by diagnostic PCR and Southern blot analysis (Figure S1 in File S1). Under *CTR4* promoter induction conditions with a copper chelator (bathocuproinedisulphonate [BCS]), all P*_CTR4_*:*ECM16*, P*_CTR4_*:*NOP14,* P*_CTR4_*:*HSP10*, and P*_CTR4_*:*MGE1* strains grew normally like the WT strain (Fig. 5B). However, under *CTR4* promoter repression conditions with CuSO_4_, all of the *CTR4* promoter replacement strains exhibited severe growth defects (Fig. 5B), indicating that Ecm16, Nop14, Hsp10, and Mge1 were truly essential proteins for the growth of *C. neoformans*.

### KR-72 induced MGE1, which is required for DNA damage repair and genotoxic stress response

**Figure 6:**
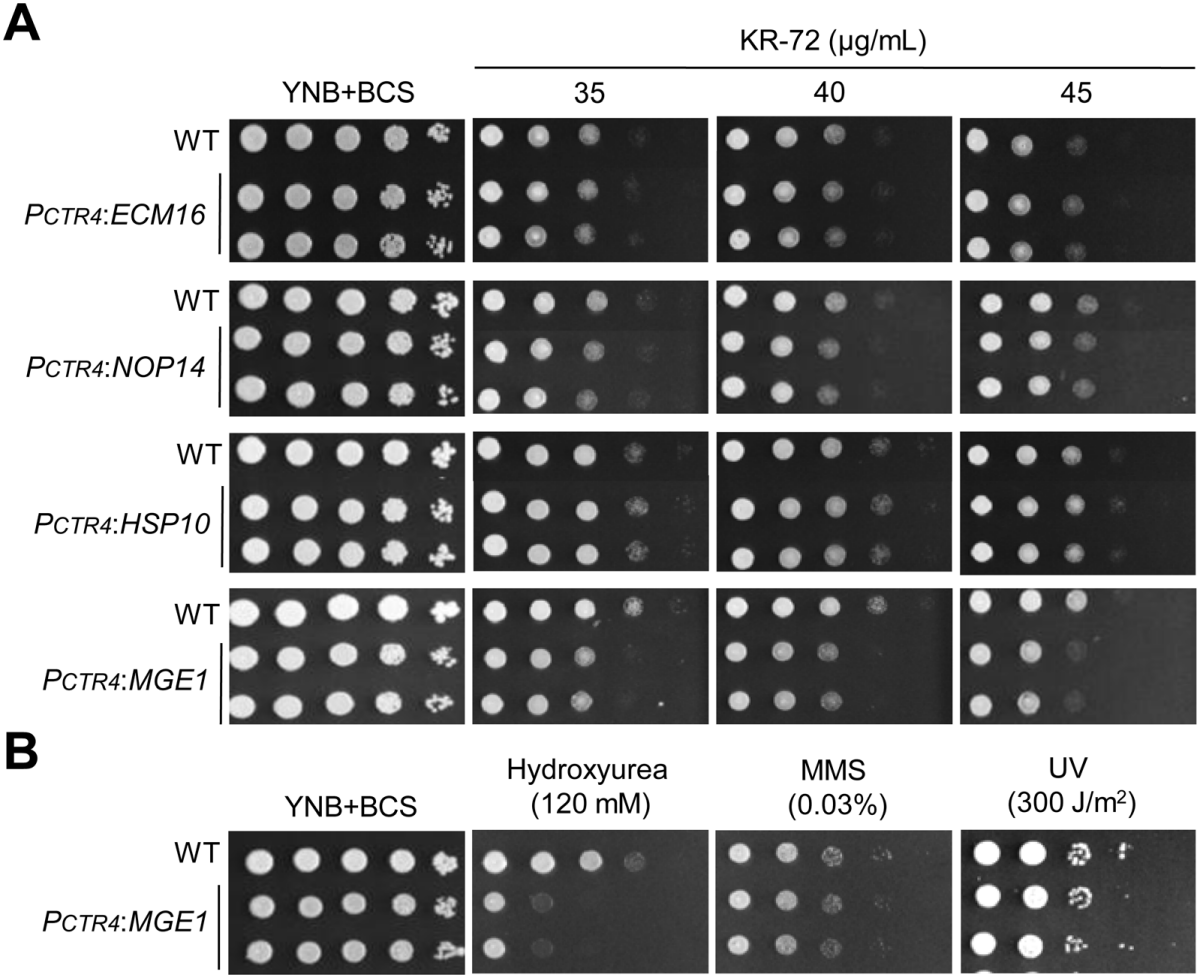
Overexpression of *MGE1* promoted cell lethality and susceptibility to genotoxic stresses in the presence of KR-72. (a) WT H99, P*_CTR4_:ECM16* (YSB2596 and YSB2597), P*_CTR4_:HSP10* (YSB2688 and YSB2689), P*_CTR4_:NOP14* (YSB2604 and YSB2605), and P*_CTR4_:MGE1* (YSB3172 and YSB3173) strains were grown in liquid YNB medium at 30°C overnight, 10-fold serially diluted (1 to 10^4^ dilutions) in sterile distilled water. Then cell suspensions (3 µL) of each strain were spotted onto solid YNB agar medium containing the indicated concentration of KR-72. Cells were incubated at 30°C for 3 days and then photographed. (b) The genotoxic response of the *MGE1* overexpression strain in *C. neoformans*. WT H99 and P*_CTR4_*:*MGE1* (YSB3172 and YSB3173) strains were grown in liquid YNB medium at 30°C overnight and 10-fold serially diluted (1 to 10^4^ dilutions) in dH_2_O. Cell suspensions (3 µL) of each strain were then either spotted onto solid YNB agar medium containing the indicated concentration of hydroxyurea (HU) and methyl methanesulfonate (MMS) or exposed to UV. Cells were incubated at 30°C for 3 days and then photographed.

One major benefit of using the *CTR4* promoter system is that genes driven by the *CTR4* promoter are highly overexpressed in the presence of BCS [28]. By utilizing this property, we examined whether the overexpression of the four essential genes conferred a certain level of resistance to KR-72. If so, this gene(s) could be the direct target of KR-72, as the overexpression of *ERG11* significantly confers azole drug resistance in yeast [29]. However, we found that the induction of *ECM16, HSP10, NOP14* and *MGE1* did not enhance any resistance to KR-72 (Fig. 6A). Under BCS-induction conditions, P*_CTR4_*:*ECM16*, P*_CTR4_*:*NOP14* and P*_CTR4_*:*HSP10* strains were as resistant to KR-72 as the wild-type strain (Fig. 6A), suggesting that Ecm16, Nop14, and Hsp10 could be just indirectly regulated by KR-72. However, this is not surprising because KR-72 treatment upregulated many other essential genes in these categories (Table 1).

Notably, however, the P*_CTR4_*:*MGE1* strain was more sensitive to KR-72 than the wild-type strain under BCS-induction conditions (Fig. 6A). This observation implies that KR-72 treatment promotes the expression of *MGE1*, which may subsequently reduce the cell viability of *C. neoformans*. This was an unexpected finding because we had originally expected that an increase in the expression of *MGE1*, which encodes a mitochondrial molecular co-chaperone, by KR-72 could occur because of a compensatory effect. Yeast Mge1 is structurally and functionally homologous to bacterial GrpE, which is a nucleotide exchange factor as well as a heat shock protein [30]. In *S. cerevisiae*, Mge1 is an essential nucleotide exchange factor involved in recycling mHsp70. The Mge1/mHsp70 complex along with Pam16 and Pam18 regulate important mitochondrial proteins and folding [31]–[33]. Mge1 also acts as a thermosensor and an oxidative sensor. In response to high temperature and oxidative stress, Mge1 does not form a dimer and interact with mHsp70 [34], [35]. Surprisingly, the overexpression of *MGE1* and its null mutation are lethal to cells. *MGE1* was identified to be involved in cell cycle progression through an extensive overexpression screen [36], and its overexpression appeared to affect DNA replication. To examine whether the lethal effects of *MGE1* overexpression in *C. neoformans* resulted from altered DNA replication and cell cycle progression, we tested the genotoxic sensitivity of the P*_CTR4_*:*MGE1* strain to hydroxyurea (HU; a ribonucleotide reductase inhibitor that blocks DNA synthesis), methyl methanesulfonate (MMS; DNA-alkylating agent that induces DNA double strand breaks), and UV irradiation (inducing pyrimidine dimers). The P*_CTR4_*:*MGE1* strain exhibited highly increased sensitivity to HU, but not MMS and UV (Fig. 6B), suggesting that Mge1 could be involved in DNA damage repair and genotoxic stress response. Therefore, consistent with the previous studies in yeast, the present study indicates that the toxic effects of *MGE1* overexpression result from perturbed cell cycle progression and that a balanced expression of *MGE1* may be critical for the viability of fungi.

**Figure 7:**
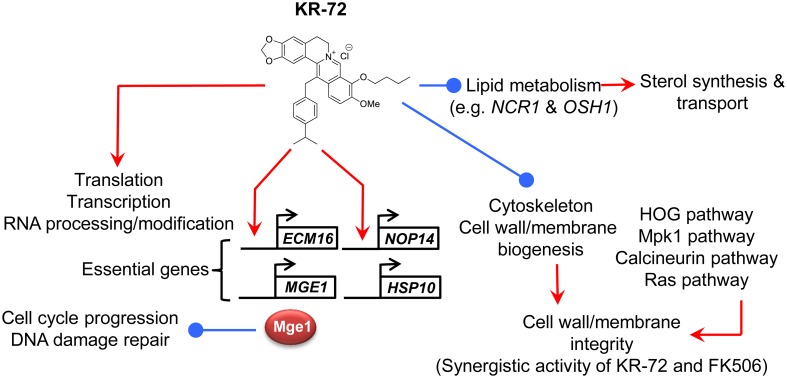
The proposed antifungal mode of action for KR-72. KR-72 treatment generally upregulates expression of genes involved in translation and transcription, while it downregulates expression of genes involved in lipid metabolism, sterol transport, cytoskeleton, and cell membrane/wall biogenesis. Sterol transport is reduced by KR-72 by reduced expression of *NCR1*. KR-72 exhibits highly synergistic antifungal activity with the calcineurin inhibitor FK506. A number of essential genes, including *ECM16, HSP10, NOP14*, and *MGE1*, are upregulated by KR-72 and the four genes were confirmed to be required for the viability of *C. neoformans*. Particularly, KR-72 treatment induced *MGE1* expression, which subsequently renders cells to be more vulnerable to genotoxic stresses.

In conclusion, KR-72, 9-*O*-butyl-13-(4-isopropylbenzyl)berberine, exhibited antifungal activity by modulating diverse biological processes in fungi and showed synergistic interaction with FK506. The proposed mode of action for KR-72 is summarized in Figure 7.

## Materials and Methods

### Preparation of 9-O-butyl-13-(4-isopropylbenzyl)berberine, KR-72

Compound KR-72 was prepared as previously reported with modifications [4]. To an aqueous 5N NaOH solution (25 mL) of berberine chloride (5.0 g), acetone (5 mL) was added dropwise. The reaction solution was allowed to stir at room temperature for 1 h. The reaction mixture was filtered, washed (80% MeOH, 100 mL), and then dried in vacuum to yield 4.6 g (86% yield) of crude 8-acetonyldihydroberberine as a yellow solid. ^1^H-NMR (300 MHz, DMSO-*d*_6_) *δ* 7.25 (s, 1H), 6.86 (d, *J* = 8.4 Hz, 1H), 6.75 (s, 1H), 6.71 (d, *J* = 8.4 Hz, 1H), 5.98–6.03 (m, 3H), 5.17–5.24 (m, 1H), 3.75 (s, 6H), 3.10–3.32 (m, 2H), 2.93 (dd, *J* = 6.5, 14.6 Hz, 1H), 2.61–2.84 (m, 2H), 2.30 (dd, *J* = 4.6, 14.6 Hz, 1H), 2.03 (s, 3H). 8-Acetonyldihydroberberine (4.0 g) dissolved in acetonitrile was treated with NaI (1.9 g) and 4-isopropylbenzyl bromide (2.6 mL) at 80°C for 5 h. The resulting reaction mixture was concentrated and purified by column chromatography on SiO_2_ to yield 2.7 g (57% yield) of 13-(4-Isopropylbenzyl)berberine as a yellow solid. ^1^H-NMR (300 MHz, DMSO-*d*_6_) *δ* 10.03 (s, 1H), 8.10 (d, *J* = 9.4 Hz, 1H), 7.79 (d, *J* = 9.4 Hz, 1H), 7.23 (d, *J* = 8.0 Hz, 2H), 7.17 (s, 1H), 7.08 (d, *J* = 8.0 Hz, 2H), 6.98 (s, 1H), 6.08 (s, 2H), 4.87 (br s, 2H), 4.69 (br s, 2H), 4.11 (s, 3H), 4.02 (s, 3H), 3.10–3.20 (m, 2H), 2.80–2.95 (m, 1H), 1.19 (d, *J* = 6.9 Hz, 6H). The pyrolysis of 13-(4-isopropylbenzyl)berberine (2.5 g) was performed at 170°C in DMF for 2 h. The resulting reaction mixture was evaporated and purified by column chromatography on SiO_2_ to yield 1.8 g (76% yield) of 13-(4-isopropylbenzyl)berberrubine as a red solid. ^1^H-NMR (300 MHz, DMSO-*d*_6_) *δ* 9.63 (s, 1H), 7.55 (d, *J* = 8.6 Hz, 1H), 7.22 (d, *J* = 8.0 Hz, 2H), 7.04–7.13 (m, 3H), 6.93 (s, 1H), 6.76–6.82 (m, 1H), 6.04 (s, 2H), 4.65 (br s, 2H), 4.48 (s, 2H), 3.84 (s, 3H), 3.04–3.15 (m, 2H), 2.83–2.92 (m, 1H), 1.19 (d, *J* = 6.9 Hz, 6H). 13-(4-Isopropylbenzyl)berberrubine (1.5 g) was dissolved in acetonitrile and reacted with iodobutane (1.1 mL) at 120°C for 16 h. The product was purified by column chromatography on SiO_2_ and then treated with AgCl (0.4 g) in hot MeOH (50°C, 30 mL) for 2 hr to afford 0.9 g (65% yield) of KR-72 as a yellow powder. ^1 ^H-NMR (300 MHz, DMSO-*d*_6_) *δ* 9.89 (s, 1H), 8.09 (d, *J* = 9.4 Hz, 1H), 7.78 (d, *J* = 9.4 Hz, 1H), 7.23 (d, *J* = 8.0 Hz, 2H), 7.17 (s, 1H), 7.08 (d, *J* = 8.0 Hz, 2H), 6.99 (s, 1H), 6.08 (s, 2H), 4.90 (br s, 2H), 4.70 (s, 2H), 4.32 (t, *J* = 6.8 Hz, 2H), 4.01 (s, 3H), 3.11–3.19 (m, 2H), 2.83–2.93 (m, 1H), 1.84–1.93 (m, 2H), 1.47–1.59 (m, 2H), 1.19 (d, *J* = 6.9 Hz, 6H), 0.99 (t, *J* = 7.3 Hz, 3H); LRMS (+ESI) 510.3 [M^+^] (calculated for C_33_H_36_NO_4_ 510.3 [M^+^]).

### *C. neoformans* strains

The *C. neoformans* strains used in this study are listed in Table S1 in File S1. *C. neoformans* strains were cultured and maintained on yeast extract-peptone-dextrose (YPD) medium unless indicated otherwise.

### Total RNA isolation

The total RNA for the DNA microarray analysis was isolated as follows. The wild-type (WT) H99 strain was grown in YPD (yeast extract-peptone-dextrose) medium at 30°C for 16 h, subcultured into fresh YPD medium (1∶20 dilution), and further incubated at 30°C for 4–5 h until the culture reached an optical density (OD) of approximately 1 at 600 nm (OD_600 nm_ = 1.0). KR-72 was added to the exponentially grown culture at a final concentration of 1 mg/L, which is a sub-lethal concentration for the *C. neoformans* H99 strain (MIC = 8 mg/L), and further incubated at 30°C for 60 min. At each time point (0 min, 30 min, and 60 min), a portion of the cells (50 mL culture) was sampled, precipitated by centrifugation, frozen in liquid nitrogen, and lyophilized overnight. Total RNA was isolated using TRIzol reagent as previously described [18] and further purified using the RNeasy Plus mini kit (Qiagen, USA). The quality of purified total RNA was confirmed using the Agilent 2100 Bioanalyzer (data not shown). Three independent cultures were used to isolate total RNA for DNA microarray analysis. To prepare reference RNA (Cy3-labelled RNA), all of total RNA samples from drug-treated or non-treated cells were pooled at equal concentrations (pooled reference RNA).

### DNA microarray and data analysis

For control (pooled reference RNA samples) and test RNA samples (RNA samples corresponding to specific time points after KR-72 treatment), the synthesis of target cRNA probes and hybridization were performed using Agilent’s Low Input QuickAmp Labeling Kit (Agilent Technology, USA) according to the manufacturer’s instructions. Briefly, 50 ng total RNA was added with T7 promoter primer mix and incubated at 65°C for 10 min. cDNA master mix (5× first strand buffer, 0.1 M DTT, 10 mM dNTP mix, RNase-Out, and MMLV-RT) was prepared and added to the reaction mixture. The samples were incubated at 40°C for 2 h, and then the reverse transcription (RT) and dsDNA synthesis was terminated by incubating at 70°C for 10 min. The transcription master mix was prepared using the manufacturer’s protocol (4x transcription buffer, 0.1 M DTT, NTP mix, 50% PEG, RNase-Out, inorganic pyrophosphatase, T7-RNA polymerase and cyanine 3/5-CTP). Transcription of dsDNA was performed by adding the transcription master mix to the synthesized dsDNA and incubating the samples at 40°C for 2 h. Amplified and labelled cRNA was purified using an RNase mini column (Qiagen) according to the manufacturer’s protocol. Labelled cRNA targets were quantified using an ND-1000 spectrophotometer (NanoDrop Technologies, Inc., Wilmington, DE). After determining the labelling efficiency, the cyanine 3-labelled RNA control and cyanine 5-labelled cRNA target samples were mixed, and the fragmentation of cRNA was performed by adding 10× blocking agent and 25× fragmentation buffer and incubating the mixture at 60°C for 30 min. The fragmented cRNA was resuspended with 2× hybridization buffer and directly pipetted onto assembled *C. neoformans* 3×20 K microarray (MYcroarray). The arrays were hybridized at 65°C for 17 h using an Agilent hybridization oven (Agilent Technology) and then washed following the manufacturer’s protocol (Agilent Technology). Finally, microarrays were spin-dried and stored in the dark until they were scanned. The hybridization images were analysed by the Agilent DNA microarray scanner (Agilent Technology) and data quantification was performed using Agilent Feature Extraction software 9.3.2.1 (Agilent Technology). The average fluorescence intensity for each spot was calculated and local background was subtracted. Data normalization and the selection of fold-changed genes were performed using GeneSpring GX 7.3.1 (Agilent Technology). Genes were filtered by removing flag-out genes in each experiment. Intensity-dependent normalization (LOWESS) was performed such that the ratio was reduced to the residual of the LOWESS fit of the intensity vs. ratio curve. The averages of normalized ratios were calculated by dividing the average of the normalized signal channel intensity by that of the normalized control channel intensity. The entire microarray data sets were deposited to Gene Expression Omnibus (GEO, www.ncbi.nlm.nih.gov/geo/) with accession GSE 55337.

### Construction of the *CTR4* promoter replacement strains

To replace the native *MGE1* promoter with the copper-regulated *CTR4* promoter, we constructed *MGE1* promoter replacement cassettes. In the first round of PCR, the 5′– and 3′-flanking regions of *MGE1* were amplified by using ExTaq polymerase (Takara) or 2×TOP simpleTM DyeMIS (Enzynomics) with the primer pairs B5575/B5576 and B5577/B5578, respectively. The *NAT-CTR4* promoter fragment in the plasmid pNAT-CTR4-2 (provided by John Perfect at Duke University) was PCR-amplified using the primer pair B354/B355. The *MGE1* promoter replacement cassette was produced by overlap PCR using combined first-round PCR products as templates and the primer pair, B5575/B5578. The overlap PCR product was purified by using Gel Sv kit (Geneall) and coated onto gold microcarrier beads (0.6 µm; BioRad) and biolistically transformed into the WT H99 strain. The same strategy described above was used to delete the *HSP10*, *NOP14*, and *ECM16* using the primers listed in Table S2 in File S1. Positive transformants were selected on YPD medium containing nourseothricin (100 mg/L) and initially screened by diagnostic PCR. The correct genotype of each *CTR4* promoter replacement strain was confirmed by Southern blot analysis as previously described [37]. Each gene-specific probe was generated by PCR with the primers listed in Table S2 in File S1.

### Northern blot analysis

Each strain was grown in YPD medium at 30°C for 16 h, inoculated into fresh YPD medium, and then further incubated at 30°C until an optical density of 1 at 600 nm was reached (OD_600 nm_ = 1.0). An aliquot of culture with or without treatment of KR-72 was sampled at different time points, frozen in liquid nitrogen for 30 min, and lyophilized overnight. Total RNA was isolated using Ribo-Ex (Geneall) as described previously [37]. Northern blot analysis was performed using 10 µg of total RNA per sample as previously described [37].

### Filipin fluorescence staining

The wild-type strain (H99) was incubated in YPD medium at 30°C overnight. The overnight culture was subcultured in 50 mL fresh YPD medium with OD_600 nm_ = 0.4 and further incubated about 4 hr at 30°C with shaking until it reaches to OD_600 nm_ = 1.0. A portion of the liquid culture (20 mL) was treated with an indicated concentration of KR-72 or H_2_O_2_. 5 mL of culture was sampled, and fixed. For filipin staining, cells were incubated with 12.5 mM filipin working solution (F-9765; Sigma-Aldrich) for 2 hr at room temperature. Cells were rinsed three times in phosphate buffered saline (PBS). After washing, cells were visualized by a fluorescence microscope (a Eclipse Ti-U; Nikon) and fluorescence levels were quantitatively measured by fluorescence microplate reader (Molecular devices spectra Max Gemini EM). Fluorescence was measured at the range of 485 to 515 nm wavelength and normalized by OD_595 nm_, which reflects cell density per sample. Three independent experiments with triplicates were performed.

#### Stress sensitivity and antifungal susceptibility test

Cells were incubated in 2 mL YPD medium at 30°C overnight, 10-fold serially diluted (1 to 10^4^ dilutions) in sterile distilled water, and spotted (3 µL) onto solid YPD medium containing the indicated concentrations stress reagents and antifungal drugs. Each plate was incubated for 2–5 days and photographed during the incubation period.

The fractional inhibitory concentration (FIC) index was calculated as described before [38] with the following modification. To determine interaction between two drugs, FICs and indexes were calculated by the equation: FIC index = FIC_A_+FIC_B_, where FIC_A_ is defined as the MIC_50_ of the drug A in combination with the drug B (at the range of MIC_50_) divided by the MIC_50_ of the drug A when used alone and FIC_B_ is calculated in the same way. Based on the FIC index, drug interactions were classified as synergistic (FIC≤0.5), no interactions (0.5<FIC≤4.0), and antagonistic (FIC>4.0) as suggested by Johnson et al [38].

## Supporting Information

File S1 **Figure S1,** Construction of *CTR4* promoter replacement strains. (A-D) The overlap PCR transformation strategy for *CTR4* promoter replacement strains with gene specific primers with were listed in Table S2 in File S1. The lower diagrams represent the *CTR4* promoter replacement alleles. Then genomic DNA was digested with specific enzymes for checking replacement of *CTR4* promoter and electrophoresed on a 1% TAE agarose gel. Transfer, hybridization and autoradiography were performed as followed by Southern blot hybridization using a gene-specific probe with was radioactively ^32^P-labeled. **Table S1,** *C. neoformans* strains used in this study. **Table S2,** Primers used in this study. **Table S3,** List of KR-72 responsive genes in *C. neoformans*. **Table S4,** List of KR-72 responsive genes, whose expression changes were more than 1.5 fold. **Table S5,** List of *C. neoformans* genes downregulated by KR-72. **Table S6,** List of *C. neoformans* genes upregulated by KR-72.
(ZIP)
