# Supplementary material for: 9-O-butyl-13-(4-isopropylbenzyl)berberine, KR-72, Is a Potent Antifungal Agent That Inhibits the Growth of Cryptococcus neoformans by Regulating Gene Expression
Source: PLoS One. 2014 Oct 10;9(10):e109863. doi: 10.1371/journal.pone.0109863 (PMC4193857; doi:10.1371/journal.pone.0109863)
Supplement: File S1 — Figure S1, Construction of CTR4 promoter replacement strains. (A-D) The overlap PCR transformation strategy for CTR4 promoter replacement strains with gene specific primers with were listed in Table S2 in File S1. The lower diagrams represent the CTR4 promoter replacement alleles. Then genomic DNA was digested with specific enzymes for checking replacement of CTR4 promoter and electrophoresed on a 1% TAE agarose gel. Transfer, hybridization and autoradiography were performed as followed by Southern blot hybridization using a gene-specific probe with was radioactively 32P-labeled. Table S1, C. neoformans strains used in this study. Table S2, Primers used in this study. Table S3, List of KR-72 responsive genes in C. neoformans. Table S4, List of KR-72 responsive genes, whose expression changes were more than 1.5 fold. Table S5, List of C. neoformans genes downregulated by KR-72. Table S6, List of C. neoformans genes upregulated by KR-72. (ZIP) [file pone.0109863.s001.zip › File S1/Table S1.docx]

Table S1. *C. neoformans* strains used in this study

| Strain | Genotype | Parent | Reference |
| --- | --- | --- | --- |
| H99 | *MATα* |  | [[1](#_ENREF_1)] |
| YSB2594 | *MATα P_CTR4_:ECM16 NAT* | H99 | This study |
| YSB2596 | *MATα P_CTR4_:ECM16 NAT* | H99 | This study |
| YSB2597 | *MATα P_CTR4_:ECM16 NAT* | H99 | This study |
| YSB2598 | *MATα P_CTR4_:ECM16 NAT* | H99 | This study |
| YSB2599 | *MATα P_CTR4_:ECM16 NAT* | H99 | This study |
| YSB3172 | *MATα P_CTR4_:MGE1 NAT* | H99 | This study |
| YSB3173 | *MATα P_CTR4_:MGE1 NAT* | H99 | This study |
| YSB3174 | *MATα P_CTR4_:MGE1 NAT* | H99 | This study |
| YSB2607 | *MATα P_CTR4_:HSP10 NAT* | H99 | This study |
| YSB2608 | *MATα P_CTR4_:HSP10 NAT* | H99 | This study |
| YSB2688 | *MATα P_CTR4_:HSP10 NAT* | H99 | This study |
| YSB2689 | *MATα P_CTR4_:HSP10 NAT* | H99 | This study |
| YSB2690 | *MATα P_CTR4_:HSP10 NAT* | H99 | This study |
| YSB2602 | *MATα P_CTR4_:NOP14 NAT* | H99 | This study |
| YSB2604 | *MATα P_CTR4_:NOP14 NAT* | H99 | This study |
| YSB2605 | *MATα P_CTR4_:NOP14 NAT* | H99 | This study |
| YSB2606 | *MATα P_CTR4_:NOP14 NAT* | H99 | This study |

[1] J.R. Perfect, N. Ketabchi, G.M. Cox, C.W. Ingram, and C.L. Beiser, Karyotyping of *Cryptococcus neoformans* as an epidemiological tool. J. Clin. Microbiol. 31 (1993) 3305-9.
